# Transplantation of mesenchymal stem cells and their derivatives effectively promotes liver regeneration to attenuate acetaminophen-induced liver injury

**DOI:** 10.1186/s13287-020-01596-9

**Published:** 2020-02-27

**Authors:** Chenxia Hu, Lingfei Zhao, Zhongwen Wu, Lanjuan Li

**Affiliations:** 1 Collaborative Innovation Center for the Diagnosis and Treatment of Infectious Diseases, State Key Laboratory for the Diagnosis and Treatment of Infectious Diseases, The First Affiliated Hospital, School of Medicine, Zhejiang University, Hangzhou, China; 2 National Clinical Research Center for Infectious Diseases, The First Affiliated Hospital, School of Medicine, Zhejiang University, Hangzhou, Zhejiang People’s Republic of China; 3 Kidney Disease Center, First Affiliated Hospital, College of Medicine, Zhejiang University, Hangzhou, Zhejiang People’s Republic of China; 4 Key Laboratory of Kidney Disease Prevention and Control Technology, Zhejiang Province, Hangzhou, Zhejiang People’s Republic of China; 5 Institute of Nephrology, Zhejiang University, Hangzhou, Zhejiang People’s Republic of China

**Keywords:** Acetaminophen, Liver injury, Liver transplantation, Mesenchymal stem cell, Liver failure

## Abstract

Acetaminophen (APAP)-induced injury is a common clinical phenomenon that not only occurs in a dose-dependent manner but also occurs in some idiosyncratic individuals in a dose-independent manner. APAP overdose generally results in acute liver injury via the initiation of oxidative stress, endoplasmic reticulum (ER) stress, autophagy, liver inflammation, and microcirculatory dysfunction. Liver transplantation is the only effective strategy for treating APAP-induced liver failure, but liver transplantation is inhibited by scarce availability of donor liver grafts, acute graft rejection, lifelong immunosuppression, and unbearable costs. Currently, *N*-acetylcysteine (NAC) effectively restores liver functions early after APAP intake, but it does not protect against APAP-induced injury at the late stage. An increasing number of animal studies have demonstrated that mesenchymal stem cells (MSCs) significantly attenuate acute liver injury through their migratory capacity, hepatogenic differentiation, immunoregulatory capacity, and paracrine effects in acute liver failure (ALF). In this review, we comprehensively discuss the mechanisms of APAP overdose-induced liver injury and current therapies for treating APAP-induced liver injury. We then comprehensively summarize recent studies about transplantation of MSC and MSC derivatives for treating APAP-induced liver injury. We firmly believe that MSCs and their derivatives will effectively promote liver regeneration and liver injury repair in APAP overdose-treated animals and patients. To this end, MSC-based therapies may serve as an effective strategy for patients who are waiting for liver transplantation during the early and late stages of APAP-induced ALF in the near future.

## Introduction

Acetaminophen (APAP) previously served as a safe and effective antipyretic and analgesic, but overdose rapidly triggers acute liver injury, which is a common clinical phenomenon with restricted treatment options. In general, doses of APAP ranging from 1 to 4 g/day are considered to be clinically safe, while a single overdose ingestion due to suicide or unintentional overdose exceeding 15 to 25 g may cause severe and lethal liver injury [1, 2]. APAP-induced liver injury, which is the most common cause of acute liver failure (ALF) in the USA, occurs not only in a dose-dependent manner but also in a dose-independent manner in some idiosyncratic individuals [3]. Delayed medical attention further makes liver injury hard to treat, and APAP overdose consequently leads to ALF because the treatable stage is missed. Finally, liver transplantation serves as the only effective strategy to rescue patients and animals with APAP-induced liver failure [4]. However, liver transplantation is inhibited by scarce availability of donor liver grafts, acute graft rejection, lifelong immunosuppression and unbearable costs [5]. Because of these disadvantages, novel therapies targeting liver regeneration may benefit APAP-induced liver injury to completely compensate for the loss of liver function. Current investigations have suggested that APAP overdose-induced liver regeneration is regulated by various factors, including growth factors, cytokines, angiogenic factors, and several signaling pathways, in a dose-dependent manner. In addition to symptomatic patient care, *N*-acetylcystenie (NAC) was reported to exert protective effects even after depletion of hepatocellular glutathione (GSH) and generation of mitochondrial reactive oxygen species (ROS) and peroxynitrite. Furthermore, superfluous NAC in the liver supplies adenosine triphosphate (ATP) production and serves as an energy substrate for the Krebs cycle to improve mitochondrial function [6]. Although pharmacological intervention with NAC effectively restores liver function at the early stage of APAP intake, it does not protect against APAP-induced injury in late-presenting patients. Patients who do not receive NAC treatment in a timely manner will develop drug-induced ALF. Patients with APAP overdose-induced ALF at an early stage may only present marked metabolic acidosis and significantly increased levels of lactate, and they also present mildly elevated levels of transaminases and minimal coagulopathy. However, patients with APAP overdose-induced ALF at later stages may develop severe liver injury, as shown by significantly upregulated levels of transaminases and marked coagulopathy [7].

Mesenchymal stem cells (MSCs) can be obtained from various tissues, such as bone marrow, adipose tissue, and umbilical cord blood [8]. These multipotent stem cells undergo self-renewal and can be differentiated into various somatic cells, such as adipose cells, osteocytes, chondrocytes, and hepatocyte-like cells (HLCs) [9]. However, MSCs only acquire liver-specific markers and functions of immature hepatocytes after hepatogenic differentiation in vitro. In addition, Sato et al. demonstrated that MSCs rarely undergo differentiation into HLCs after homing to injured liver [10]. MSCs participate in promoting liver regeneration and repairing liver injury after migrating into injured tissues, undergoing hepatogenic differentiation, reducing apoptosis of hepatocytes, promoting hepatocyte proliferation, and exerting anti-inflammatory and immunoregulatory effects in human and rodent models [8]. Multiple cytokines, including interleukin (IL)-1β, IL-6, IL-8, monocyte chemoattractant protein-1 (MCP-1), growth-regulated protein (GRO), tumor necrosis factor (TNF)-α, transforming growth factor (TGF)-β1, platelet-derived growth factor (PDGF), vascular endothelial growth factor (VEGF), and stromal cell-derived factor 1 (SDF-1), are secreted by injured liver tissues and effectively induce the migration of MSCs into injured liver sites [11]. In addition, transplantation of MSCs significantly upregulates the expression of insulin-like growth factor 1 (IGF-1) in the livers of recipients, and MSCs have been traced up to 14 days after transplantation in vivo [12]. In a coculture system consisting of MSCs and LX-2 cells, osteoprotegerin (OPG) was significantly upregulated compared with that of LX-2 and MSC single-culture control groups. Moreover, the expression of OPG was lower in the LX-2 single-culture control group than in the other two groups, which indicates that OPG plays a critical role in the chemotactic effect of MSCs [13]. A large number of necrotic hepatocytes or cholangiocytes further upregulate the infiltration of leucocytes, monocytes, activated Kupffer cells (KCs), liver sinusoidal endothelial cells (LSECs), and hepatic stellate cells (HSCs) [14]. Furthermore, MSCs take part in liver regeneration by releasing cytokines such as hepatocyte growth factor (HGF), fibroblast growth factor (FGF), IL-6, fibrinogen, and TGF-α, which effectively enhance hepatocyte proliferation [15]. HGF and epidermal growth factor (EGF) induce hepatic progenitor cell proliferation and differentiation for liver regeneration [16]; moreover, VEGF-mediated angiogenesis via recruitment of rat LSEC progenitor cells serves as another important mechanism for liver regeneration [17]. Likewise, in vivo transplantation of conditioned medium (CM) from MSCs effectively inhibited hepatocellular death and increased liver regeneration in ALF models [18].

In this review, we comprehensively discuss the mechanisms of APAP overdose-induced liver injury and current therapies for treating APAP-induced liver injury. We then comprehensively summarize recent studies about transplantation of MSCs and MSC derivatives for treating APAP-induced liver injury. We firmly believe that MSCs and their derivatives will effectively promote liver regeneration and liver injury repair in APAP overdose-treated animals and patients.

### In vivo and in vitro APAP overdose models

Only a very high dose (1 g/kg) of APAP induces minor liver injury in rats; they are not sensitive to APAP overdose, since they have lower levels of mitochondrial protein binding, which inhibits the progression of mitochondrial damage and oxidative stress [19]. To mimic APAP overdose in humans, 200 to 400 mg/kg APAP is utilized to induce liver injury in fasted mice [19]. APAP (500 to 600 mg/kg) is administered to fed mice, but this kind of model will generate more variable liver injury [19]. However, a minor difference exists between humans and mice, as APAP-induced hepatotoxicity peaks at 12–24 h in mice and peaks at 24–72 h in humans [20]. Because of the similar dose and mechanisms of APAP-induced toxicity in humans and mice, mice serve as the best available models for APAP overdose-induced liver injury [21]. In addition to in vivo experiments with APAP-treated animal models, in vitro cultures of human hepatocytes are valuable and are the preferred tools to investigate the cellular events related to APAP hepatotoxicity. In cultured hepatocytes, 5 to 40 mM APAP is utilized to induce hepatoxicity. However, there are pronounced interspecies differences among different hepatocytes, including mouse hepatocytes, rat hepatocytes, and human hepatocytes [22]. Cultured mouse hepatocytes are more sensitive to APAP overdose than human hepatocytes, as McGill et al. showed that *NAC* was not effective beyond 2 h after APAP exposure in cultured mouse hepatocytes [23]. Multiple studies demonstrated that stem cell-derived hepatocytes displayed similar transcriptional profiles as those of primary hepatocytes, and stem cell-derived hepatocytes are used to test APAP-induced hepatocyte injury in vitro. Human-induced pluripotent stem cell-derived hepatocytes (hiPSC-Heps) and primary cryopreserved human hepatocytes (cryo-hHeps) hold great potential as unlimited cell sources for APAP toxicity testing in drug discovery research [24]. hiPSC-Heps in suspension display enhanced enzymatic inducibility and metabolic function, which can further replace primary human hepatocytes [25]. Thus, stem cell-derived hepatocytes gradually became a substitute and overcame the limited number of primary hepatocytes. However, these hepatocyte-like cells have poor enzyme inducibility and immature liver functions, rapidly lose liver-specific functions and cannot proliferate under in vitro culture conditions [26]. To further enrich the number of functional hepatocytes, multiple liver cell lines derived from human hepatomas are utilized in APAP-induced hepatoxicity studies. The infinite growth capacity provides an unlimited supply of proliferating cells, and these cell lines only express reduced levels of biotransformation enzymes, especially cytochrome P450 enzymes (CYPs), compared to those of primary hepatocytes [27]. Intriguingly, HepaRG cells were reported to express similar levels of metabolizing enzymes and comparably transport drugs when compared to those of primary human hepatocytes [28]. In response to APAP-induced toxicity, HepaRG cells subsequently initiate peroxynitrite formation, protein adduct formation, mitochondrial oxidative stress, and cell necrosis [29].

As mice serve as the best models in APAP-induced hepatoxicity, they are most frequently used to clarify the potential mechanisms and therapies in preclinical studies.

### The potential mechanisms of APAP-induced liver injury

The conventional dose of APAP is mainly metabolized by UDP-glucuronosyltransferase (UGT) and sulfotransferase (SULT) into nontoxic compounds that are excreted in urine, while a small part of the remaining APAP is further metabolized by CYPs into the highly reactive intermediate metabolite *N*-acetyl-p-benzoquinone imine (NAPQI) [30]. After that, GSH conjugation effectively removes the metabolites. However, the saturation of phase II metabolizing enzymes (UGT and SULT) induced by APAP overdose subsequently initiates the depletion of GSH, mitochondrial oxidative stress, mitochondrial dysfunction, and hepatocyte necrosis [31]. NAPQI interferes with mitochondrial proteins and mitochondrial complex I/II, subsequently triggering leakage of electrons from the electron transport chain (ETC) to oxygen and generation of superoxide radicals [32]. APAP alters mitochondrial functions by impairing energy metabolism and ATP/ADP ratios in multiple in vivo and in vitro models of APAP hepatotoxicity [20]. In particular, the formation of mitochondria-derived protein adducts and the generation of mitochondrial ROS lead to excessive oxidative stress and c-jun *N*-terminal kinase (JNK) activation, which exacerbate oxidative stress-induced injury [19, 33]. Moreover, JNK activation exacerbates the overexpression of mitochondrial ROS and forms a self-sustaining activation loop, which further promotes mitochondrial permeability transition pore (MPTP) opening, endonuclease release, collapse of the membrane potential, and ATP depletion [33, 34]. The MPTP further facilitates the release and translocation of mitochondrial intermembrane proteins such as endonuclease G and apoptosis-inducing factor for the initiation of DNA fragmentation and cellular necrosis [35]. On the other hand, activated p53 plays a dual role in APAP-induced hepatotoxicity, as it protects against liver injury during the injury phase of APAP hepatotoxicity by inhibiting JNK and also delays the initiation of liver repair in the regeneration phase [36, 37]. In addition to JNK, other kinases, including receptor interacting protein kinases (Ripk) 1 and 3, also play critical roles in the regulation of APAP-induced hepatocellular necrosis [38, 39]. In addition, increasing evidence has demonstrated that APAP overdose upregulates endoplasmic reticulum (ER) stress and CHOP expression, accompanied by unfolded protein response (UPR) activation [40]. In APAP overdose-treated mice, activation of p53 and downstream p21 was associated with cell cycle arrest and prolonged double-strand DNA damage [41]. These processes ultimately lead to hepatocellular necrosis, specifically in the centrilobular region. APAP-induced acute liver injury mainly triggers the initiation of cell necrosis rather than cell apoptosis [20].

Necrotic cells promote the secretion of damage-associated molecular patterns (DAMPs), including high mobility group box 1 (HMGB1) and DNA fragments, to recruit immune cells and generate inflammatory factors [42]. DAMPs subsequently upregulate the secretion of various proinflammatory cytokines, including TNF-α, IL-1β, IL-6, and IL-10, and multiple chemokines, including MCP-1, major intrinsic protein (MIP)-2, and IL-8 [43]. Necrotic hepatocytes subsequently secrete a large number of proteolytic enzymes, such as calpains, to impair neighboring hepatocytes and exacerbate APAP-induced liver injury [44].

The final stage of APAP overdose is the recovery stage, which is accompanied by compensatory hepatocellular proliferation, removal of necrotic cells, liver regeneration, and restored liver function [45]. In response to APAP, several antioxidant enzymes, such as microsomal epoxide hydrolase, heme oxygenase-1 (HO-1), NADPH:quinone oxidoreductase 1 (NQO-1), and glutamate cysteine ligase (GCL), are activated to detoxify NAPQI after activation of Nrf2 [46]. Other repair mechanisms, including antioxidants and activation of autophagy, also contribute to attenuation of APAP-induced liver injury. Autophagy also participates in maintaining cellular homeostasis by clearing damaged mitochondria and detrimental APAP protein adducts in APAP-treated animals [47]. Only when APAP-induced hepatoxicity initiates a robust liver regeneration process can liver injury and liver function be completely restored to terminate APAP-induced ALF. Bhushan et al. showed that a moderate overdose of APAP (300 mg/kg) not only induced liver injury but also triggered regression of hepatocellular damage and liver regeneration in mice. However, administration of 600 mg/kg APAP only led to continuous liver injury without liver regeneration after activation of downstream signaling mediators, including ERK1/2, AKT, c-Jun, and c-Fos [48]. In addition, activation of TGF-β signaling in perinecrotic areas is highly associated with decreased liver regeneration and hepatocyte senescence in ALF patients and mice with APAP overdose [49].

**Fig. 1:**
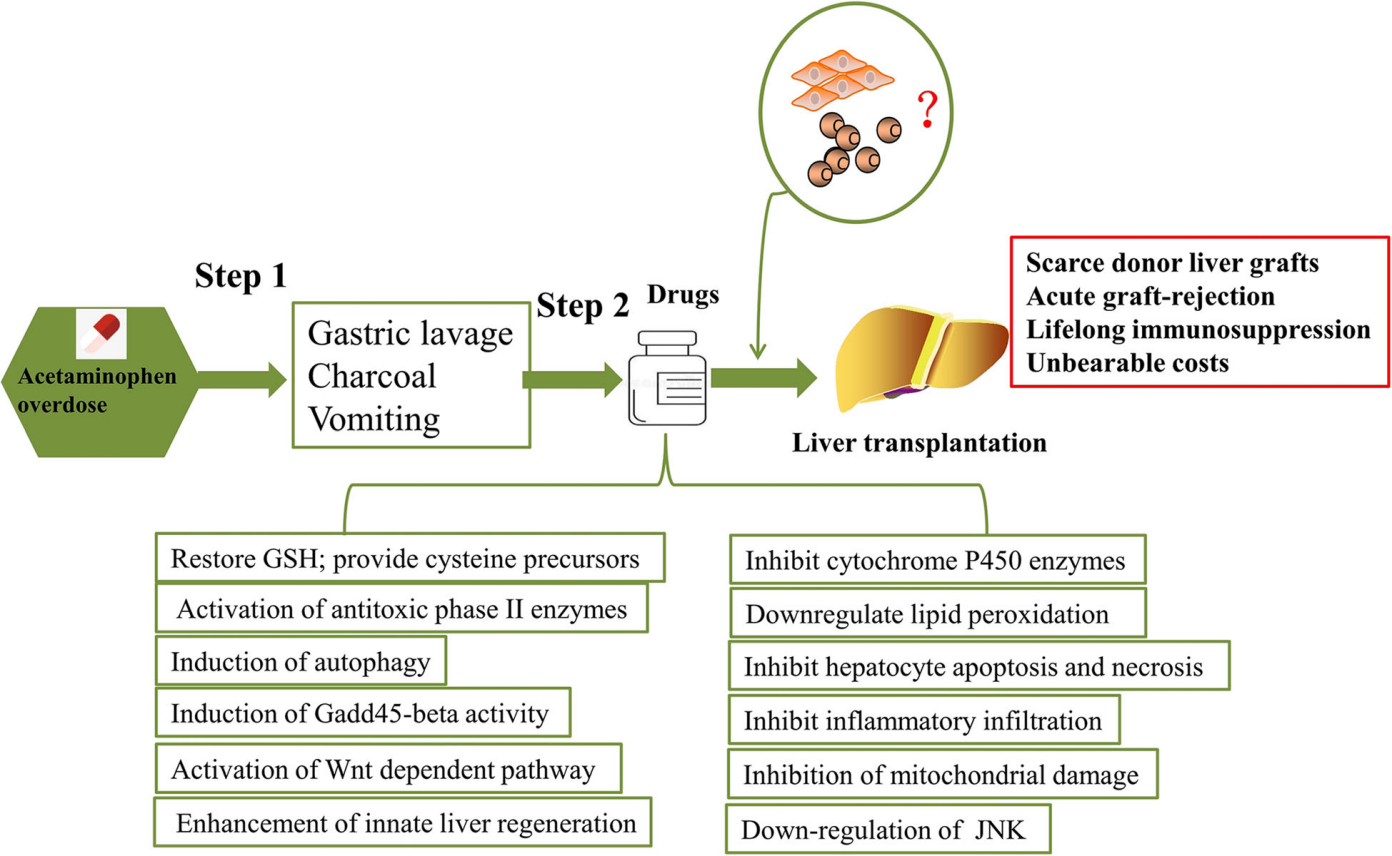
The potential mechanisms of APAP overdose metabolism in liver tissue

In conclusion, the initiation of overdose-associated APAP metabolism triggers acute liver injury after the initiation of oxidative stress, ER stress, autophagy, liver inflammation, and microcirculatory dysfunction, resulting in liver repair by inducing compensatory liver regeneration (Fig. 1).

### Current drugs targeting related pathological mechanisms to attenuate APAP-induced liver injury

When a patient with APAP overdose receives early medical attention within 4 h of APAP ingestion, gastric lavage, charcoal, and vomiting are suggested to reduce drug absorption.

Patients with persistent alanine aminotransferase (ALT) elevation, acidosis, creatinine elevation, hyperbilirubinemia, coagulopathy, and encephalopathy have the potential to have a poor prognosis, and NAC therapy should be continued for a prolonged period of time in these patients [50]. Although NAC is the recommended antioxidant to replenish GSH and scavenge mitochondria-derived ROS in APAP-overdose patients and has certain therapeutic effects, its application is inhibited by its side effects and narrow therapeutic window [6, 51]. The side effects of intravenous NAC include anaphylactic reaction, nausea, vomiting, diarrhea or constipation, fever, headache, drowsiness, and hypotension [52]. Makin et al. demonstrated that NAC effectively restored the GSH level within 24 h of APAP overdose by providing cysteine precursors [53], while late NAC treatment was harmful for recovery in APAP-treated models and was associated with further exacerbated liver injury [54].

In addition to NAC therapy, multiple studies have tried to eliminate APAP-induced hepatoxicity by targeting the relative mechanisms of APAP hepatotoxicity at the molecular level. Activation of antitoxic phase II enzymes significantly reduces the generation of NAPQI that results from APAP metabolization. For example, suppression of 5-lipoxygenase significantly reduced NAPQI production and ameliorated acute liver injury in mice via activation of the phase II enzyme SULT2A1 and inhibition of the phase I enzyme CYP 3A11 [55]. Various natural products serve as potential therapies to protect against APAP hepatotoxicity by enhancing the expression of phase II metabolic enzymes and inhibiting the expression of cytochrome P450 enzymes. Black ginseng has been suggested to act as a protective compound in APAP-treated mice and improves liver function via upregulation of GSH, downregulation of the lipid peroxidation product malondialdehyde, and inhibition of apoptosis, necrosis, and inflammatory infiltration in liver tissue [56]. Sulforaphane, a dietary isothiocyanate found in various vegetables (cauliflower, broccoli, kale, cabbage, collards, and brussels sprouts), protects against APAP-induced hepatoxicity in primary hepatocytes by inhibiting the generation of reactive oxygen species and alleviating GSH depletion and lipid peroxidation [57].

Metformin protects against APAP overdose-induced hepatotoxicity via induction of Gadd45-beta activity, downregulation of JNK, and inhibition of mitochondrial damage [58]. In addition, benzyl alcohol, which attenuates the release of inflammatory factors such as IL-1β and IL-18, protects against APAP-induced liver injury in a Toll-like receptor 4 (TLR4)-dependent manner [59]. Dabigatran etexilate, which is a thrombin inhibitor, reduces APAP-induced hepatotoxicity at an early stage but markedly exacerbates APAP-induced hepatotoxicity after 24 h [60]. Isoproterenol, a β-adrenoceptor agonist, protects against APAP-induced hepatotoxicity in both the early and late stages via a Wnt-dependent pathway [61]. The combination of high-volume plasmapheresis and standard medical therapy improves the prognosis of APAP-induced ALF patients without liver transplantation [62]. Although hepatic GSH depletion has already occurred, induction of autophagy by rapamycin at 2 h after APAP administration reduces APAP-induced liver injury, since autophagy responsibly degrades excess or aberrant long-lived cytosolic proteins and organelles [63]. Although excessive hepatic inflammation results in exacerbated liver injury, mild inflammation may contribute to liver regeneration in APAP overdose models. Moreover, enhanced innate liver regeneration effectively improves the survival rate and survival time of patients without liver transplantation [64]. Thus, strategies to enhance liver regeneration are potentially attractive for patients with APAP overdose and determine the final outcome of APAP-induced acute liver injury. Inhibition of GSK-3β significantly activates β-catenin signaling and initiates early liver regeneration even in the late time course of severe APAP overdose injury in mice [65]. Stimulation of late bile acid signaling via intervention with engineered FGF-19 led to increased liver regeneration and decreased liver injury in mice with severe APAP overdose [66]. Current studies found that several factors, such as IL-6 and VEGF, contribute to promoting liver regeneration for injury regression and liver repair. VEGF is a mitogen for angiogenesis and the restoration of the microvasculature during liver regeneration [67, 68]. Knockout of VEGFR-1 impairs generation of microvasculature, hepatocyte proliferation, and secretion of HGF and FGF in mouse models, thus decreasing the survival rate of APAP overdose ALF mice [69]. Intriguingly, there is debate about the association between the upregulation of growth factors and compensatory liver regeneration in APAP overdose models. Hughes et al. showed that the expression level of serum HGF in nonsurvivors was higher than that in survivors among APAP overdose ALF patients [70]. Although liver regeneration serves as a compensatory process to reduce hepatocyte death and liver injury, prolonged liver regeneration will ultimately induce liver cirrhosis and hepatocellular carcinoma.

**Fig. 2:**
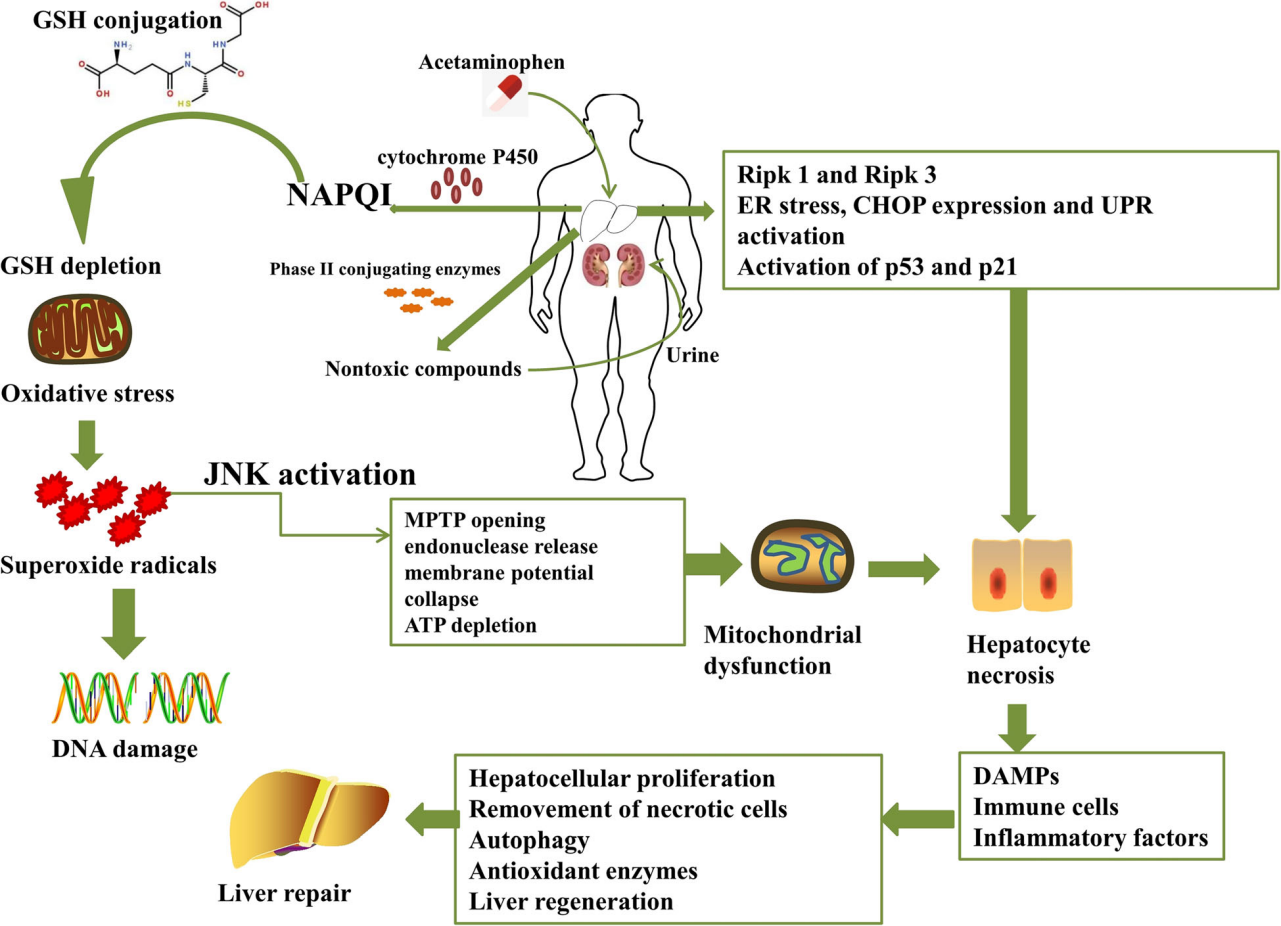
Administration of MSCs and their derivatives in APAP-induced liver injury may bridge the time gap before liver transplantation

Although these drugs or small molecules partly repair APAP-induced liver injury in the early stage, a large amount of them will exacerbate liver injury and result in ALF at the final stage. The application of liver transplantation is inhibited, as we previously mentioned; thus, further studies should address the time gap before liver transplantation (Fig. 2).

### The potential mechanisms of MSCs attenuation of acute liver injury

Although various pathophysiologic factors, including viral factors, hepatotoxic drugs, and immune injury, induce ALF in animal models and humans, hepatotoxic drugs, especially APAP, result in ALF and irreversible liver injury. ALF is currently defined as patients who develop a status with an international normalized ratio (INR) > 1.5 and any grade of hepatic encephalopathy within 26 weeks, and these patients do not have a history of liver disease [71]. Because disadvantages such as scarce availability of liver graft donors, expensive costs, and acute graft rejection limit the wide application of liver transplantation, primary hepatocyte transplantation is also limited by difficulty of expansion and poor hepatic function in vitro.

Recent studies demonstrated that MSCs reduce the serum levels of ammonia, alanine aminotransferase (ALT), aspartate aminotransferase (AST), total bilirubin (TBIL), and other inflammatory factors in ALF animal models. MSCs underwent hepatogenic differentiation after engraftment into liver tissues, and these HLCs possessed multiple liver functions, such as the secretion of albumin and alpha fetoprotein [72]. These HLCs decreased hepatocyte necrosis, enhanced liver regeneration, and improved the prognosis of ALF models. By tracking MSCs in APAP-induced ALF models, multiple studies have tried to determine whether MSCs participate in repairing liver injury through hepatogenic differentiation or by other mechanisms. MSCs labeled with near-infrared-fluorescent CuInS2.ZnS nanocrystals (CIZS-NCs) were proven to have excellent biocompatibility; moreover, CIZS-NCs had no effect on cell viability, immunophenotyping, or multipotency of MSCs in vitro. By tracking analysis, these MSCs-CIZS-NCs were initially trapped in the lungs but gradually migrated into the injured liver tissue by 2 h after transplantation. In an APAP-induced injury model, MSCs-CIZS-NCs significantly promoted liver regeneration via promoting the renewal of multiple liver cells and attenuating inflammation, necrosis, and apoptosis [73]. On the other hand, a dual labeling method was used to track stem cells in vivo in APAP-treated mice. Green fluorescence protein (GFP)-positive embryonic stem cells (ESCs) were stained with the near-infrared fluorescent lipophilic tracer 1,1-dioctadecyl-3,3,3,3-tetramethylindotricarbocyanine iodide (DiR) before transplantation into mouse models. MSCs were trapped in the spleen at 30 min after transplantation, and they gradually moved into liver tissue at 3 h after transplantation. On day 3, the DiR signal started to fade out and was no longer detectable at 2 weeks in vivo. Furthermore, GFP-positive ESCs were proven to produce albumin and were dispersed throughout the liver parenchyma after cell transplantation. However, transplanted ESCs produced very little albumin at 72 h, and few cells that were positive for GFP were also strongly positive for albumin at 2 weeks. This indicated that ESCs exert protective effects on APAP-treated mice mainly through paracrine effects rather than direct differentiation [74]. In addition, MSCs significantly attenuated liver inflammation to improve the liver functions in ALF models by downregulating TNF-α, IFN-γ, IL-1β, IL-6, and IL-10 [75]. ALF models had preserved liver functions and liver metabolic balance after MSC transplantation. The preserved liver function was due to upregulation of HO-1 and reduction of polymorphonuclear neutrophil (PMN) infiltration, subsequently resulting in enhanced liver regeneration [76]. MSCs also significantly inhibited the activation of cytotoxic T lymphocytes, natural killer (NK) cells, and NKT cells after engraftment into the injured hepatic sites via various intercellular contacts and paracrine factors [76]. Inflammatory cells such as DCs, B cells, and neutrophils impair liver function and exacerbate inflammation-induced injury in liver tissue; thus, MSC transplantation targeted to inhibit these inflammatory cells can help to improve the prognosis of ALF [77–79]. On the other hand, MSCs also exert protective roles in the elimination of immune-related liver injury by improving the activities of Tregs and M2-type macrophages but decrease the activities of T helper (Th)1 and Th17 cells in ALF models [80]. Although it is widely accepted that APAP overdose leads to depletion of GSH and excessive oxidative stress in liver tissues, MSCs effectively increase the level of antioxidants and reduce the level of nitrotyrosine to increase the survival rate of APAP-induced ALF mice [81]. In a long-term study, gradient concentrations of MSCs were proven to decrease hepatocyte necrosis and lobular neutrophilic infiltration without altering body weight, food/water consumption, clinical symptoms, urinalysis, hematology, clinical chemistry, organ weight, histopathology, or tumorigenicity in vivo [82]. Although MSCs effectively attenuate liver injury through their migratory capacity, hepatogenic differentiation, immunoregulatory capacity, and paracrine pathways in vivo, further study is needed to explore more mechanisms by which MSCs and their derivatives attenuate APAP-induced ALF. In this way, the therapeutic effects of MSCs and their derivatives can be further improved to promote liver regeneration in regenerative medicine.

### Transplantation of MSCs or MSC derivatives significantly alleviates APAP-induced liver injury

A large number of studies have focused on the mechanisms of MSCs and their derivatives in treating drug-induced liver injury, while further studies are needed to clarify the underlying mechanisms of MSCs and their derivatives in treating APAP-induced liver injury in vivo (Table 1).

**Table 1:** The underlying mechanisms of MSCs and their derivatives in treating APAP-induced liver injury in animal models

MSC source 1	MSC source 2	Dose	Model	Mechanism	Effect	Ref.
Mouse	Adipose tissue	1 × 10^6^	Mouse	Suppress cytochrome P450 activity; decrease the level of toxic nitrotyrosine; upregulate Nrf2 levels	Eliminate the severity of liver injury; increase the survival rate of ALF mice	[81]
Human	Adipose tissue	2 × 10^5^	Rat	Decrease the levels of liver isoprostanes, 8-OHG and nitrite-nitrates; preserve GSH levels; decrease levels of TNF-α, MCP-1, IL-1β, ICAM-1 and phospho-JNK	Eliminate lobular necrosis and vacuolar degeneration	[83]
Human	Umbilical cord	1 × 10^5^, 5 × 10^5^, 1 × 10^6^	Mouse	Maintain levels of glutathione and superoxide dismutase; reduce levels of TNF-α and IL-6; increase HGF levels	Increase the survival rate of ALF animals without reducing liver weight	[84]
Human	Adipose tissue	4 × 10^5^	Mouse	Prolong the retention of MSCs; alleviate the inflammatory response	Eliminate the severity of liver injury	[85]
Human	Umbilical cord	1 × 10^6^	Mouse	Maintain redox homeostasis; engraft into the injured liver tissue; reduce hepatocyte apoptosis	Reduce liver damage; enhance the survival rate of ALF mice	[86]
Human	HLCs from bone marrow MSCs	1 × 10^6^	Mouse	Reside in the injured liver site; prevent apoptosis of hepatocytes; enhance tissue rearrangement	Eliminate the severity of liver injury	[87]
Mouse	Proteome from bone marrow-derived MSCs	10 mg/ml	Mouse	Inhibit inflammation and hepatocyte necrosis	Protect against liver damage and promote liver regeneration	[88]

#### MSC transplantation for APAP-induced liver injury

APAP-induced liver injury is the most common drug-induced liver injury in clinical cases and results in a high death rate if patients are not able to receive timely treatment. Thus, it is critical to find an effective strategy to improve the prognosis of APAP-induced ALF induced even at the late phase.

A large number of MSCs in vivo will absolutely protect against APAP-induced liver injury, as shown by the downregulated expression of ALT and AST and decreased prothrombin time in APAP-induced ALF rats. MSC transplantation completely inhibited necrosis in liver tissue and diffuse vacuolar degeneration; thus, APAP-treated rats demonstrated normal levels of plasma ammonia and demonstrated no clinical encephalopathy. Salomone et al. further clarified that MSCs effectively decrease the levels of liver isoprostanes, 8-OHG, and nitrite-nitrates but preserve GSH levels, accompanied by decreased levels of TNF-α, MCP-1, IL-1β, intercellular adhesion molecule (ICAM)-1, and phospho-JNK [83]. MSCs decrease the expression of ALT, AST, and total bilirubin and increase the survival rate of ALF mice without reducing liver weight via maintaining mitochondrial metabolism (GSH and superoxide dismutase (SOD)), reducing levels of TNF-α and IL-6 while increasing HGF levels [84]. In addition, MSCs suppress cytochrome P450 activity and upregulate the Nrf2-dependent pathway, thus eliminating the severity of liver injury and increasing the survival rate of ALF mice [81]. Lipid-conjugated heparin-coated MSCs (Lip-Hep/MSCs) significantly decreased the levels of ALT and AST in serum compared to those of untreated MSCs. The lipid-conjugated heparin coating prolonged the retention of MSCs in the APAP-injured liver and alleviated the inflammatory response [85]. Moreover, heparin coating did not influence cell activity or anti-inflammatory capacity but promoted the persistent release of TGF-β1 in MSCs for liver regeneration [89].

HGF has been demonstrated to be a potent antiapoptotic and mitogenic factor in repairing liver injury. Furthermore, HGF enhances the differentiation of MSCs toward HLCs via activation of NF-кB signaling in vitro [90]. To further improve the therapeutic effects of MSCs in APAP-induced liver injury, gene modification, which can specifically express growth factors, will contribute to MSC-based regenerative medicine in ALF models. MSCs overexpressing HGF prevented liver failure and reduced mortality in rats after liver transplantation via migration into liver tissue and further hepatogenic differentiation [91]. HGF-overexpressing MSCs protected ALF mice by reducing liver damage and enhancing of the survival rate of ALF mice via maintaining redox homeostasis, such as γ-glutamylcysteine synthetase (γ-GCS), SOD, and catalase. MSCs engraft into the injured liver tissue and reduce the apoptosis of hepatocytes by upregulating antiapoptotic B cell lymphoma-2 (Bcl-2) and downregulating proapoptotic Bcl-2-associated X (Bax) and TNF-α [86].

#### MSC derivatives for APAP-induced liver injury

MSC derivatives such as MSC-derived HLCs or MSC-derived CM also contribute to liver regeneration and preserve liver functions in APAP-treated animal models.

MSCs rapidly undergo hepatogenic differentiation in vitro after coculture with specific combinations of growth factors, and these HLCs possessed hepatocyte morphology and expressed high levels of liver-specific genes in response to hepatogenic induction. Moreover, HLCs highly secrete albumin and urea, accompanied by glycogen storage, CYP activity, and intake of indocyanine green [92]. Heparinized three-dimensional (3D) scaffolds promote the generation of HLCs with high expression of late liver-specific markers, such as glucose-6-phosphatase (G6P) and CYP2B, accompanied by high levels of early liver-specific markers, including hepatocyte nuclear factor (HNF)-4, albumin, cytokeratin-18, and cytokeratin-19. HLCs that were differentiated in 3D heparinized scaffolds stored more glycogen than HLCs from nonheparinized scaffolds [93]. Multiple studies have further investigated the therapeutic effects of hepatogenic MSCs with liver-specific functions in repairing liver injury in ALF models. HLCs were found to reside in the injured liver site and prevent apoptosis of hepatocytes. Moreover, HLCs helped to maintain metabolic protein expression in the injured liver by improving the mouse liver after acute APAP intoxication [87].

MSC-derived CM or exosomes effectively rescue ALF models via multiple mechanisms. MSC-derived exosomes, which contain a variety of soluble bioactive molecules, are concentrated and ultracentrifuged from conditioned media. Intriguingly, Wu et al. demonstrated that MSC-derived exosomes promoted the conversion of hepatocytes into hepatic oval cells to promote liver regeneration [94]. Exosomes improve cell recovery and reduce cytotoxicity in APAP- and hydrogen peroxide-treated hepatocytes [95]. MSC-derived exosomes significantly inhibit APAP-induced apoptosis of hepatocytes via upregulation of Bcl-xL levels but not through modulation of oxidative stress in vitro [96]. The proteome isolated from MSCs significantly inhibited inflammation and hepatocyte necrosis in APAP-administered animals [88].

## Conclusion

APAP overdose is a universal clinical phenomenon with limited treatment options after the induction of acute liver injury and ALF, but the underlying mechanisms are still difficult to completely clarify according to current studies. APAP overdose has been proven to initiate liver injury by transformation into NAPQI and initiation of excessive oxidative stress, ER stress, autophagy, and inflammation in vivo. However, the late attention paid to APAP overdose-induced injury makes impaired liver function difficult to treat in clinics. APAP-induced liver injury initiates compensatory liver regeneration; thus, therapies that target each pathophysiological process will further improve the prognosis of APAP-induced liver failure and address the time gap before liver transplantation in ALF patients. MSCs and their derivatives effectively attenuate APAP-induced liver injury by inhibiting oxidative stress, inflammation, and hepatocyte apoptosis and promoting liver regeneration in vivo. However, a high concentration of APAP exerts cytotoxic effects in MSCs in vitro via upregulation of hydrogen peroxide, JNK/p38 signaling, and the caspase-9/caspase-3 cascade [97]. It is critical to find potential strategies to improve the therapeutic effects of MSCs to prolong the survival time of APAP overdose-treated models. To this end, MSC-based therapies will replace liver transplantation in treating early and late stages of APAP-induced ALF.

## Data Availability

All data are included in this published article.

## Abbreviations

3D: Three dimensional
ALF: Acute liver failure
ALT: Alanine aminotransferase
APAP: Acetaminophen
AST: Aspartate aminotransferase
ATP: Adenosine triphosphate
Bax: Bcl-2-associated X
Bcl-2: B cell lymphoma-2
CIZS-NCs: CuInS2.ZnS nanocrystals
CM: Conditioned medium
cryo-hHep: Cryopreserved human hepatocyte
CYPs: Cytochrome P450 enzymes
DAMPs: Damage-associated molecular patterns
EGF: Epidermal growth factor
ER: Endoplasmic reticulum
ESCs: Embryonic stem cells
ETC: Electron transport chain
FGF: Fibroblast growth factor
G6P: Glucose-6-phosphatase
GCL: Glutamate cysteine ligase
GFP: Green fluorescence protein
GRO: Growth-regulated protein
GSH: Glutathione
HGF: Hepatocyte growth factor
hiPSC-Hep: Human-induced pluripotent stem cell-derived hepatocyte
HLCs: Hepatocyte-like cells
HMGB1: High mobility group box 1
HNF: Hepatocyte nuclear factor
HO-1: Heme oxygenase-1
HSCs: Hepatic stellate cells
ICAM: Intercellular adhesion molecule
IGF-1: Insulin-like growth factor 1
IL: Interleukin
INR: International normalized ratio
JNK: c-jun *N*-terminal kinase
KCs: Kupffer cells
Lip-Hep/MSCs: Lipid-conjugated heparin-coated MSCs
LSECs: Liver sinusoidal endothelial cells
MCP-1: Monocyte chemoattractant protein-1
MIP: Major intrinsic protein
MPTP: Mitochondrial permeability transition pore
MSC: Mesenchymal stem cell
NAC: *N*-Acetylcysteine
NAPQI: *N*-Acetyl-p-benzoquinone imine
NK: Natural killer
NQO-1: NADPH:quinone oxidoreductase 1
OPG: Osteoprotegerin
PDGF: Platelet-derived growth factor
PMN: Polymorphonuclear neutrophils
Ripk: Protein kinases
ROS: Reactive oxygen species
SDF-1: Stromal cell-derived factor 1
SOD: Superoxide dismutase
SULT: Sulfotransferase
TBIL: Total bilirubin
TGF: Transforming growth factor
Th: T helper
TLR4: Toll-like receptor 4
TNF: Tumor necrosis factor
UGT: UDP-glucuronosyltransferase
UPR: Unfolded protein response
VEGF: Vascular endothelial growth factor
γ-GCS: γ-Glutamylcysteine synthetase
